# Recurrent hypoglycemia secondary to metformin toxicity in the absence of co-ingestions: a case report

**DOI:** 10.1186/s13256-018-1758-0

**Published:** 2018-08-18

**Authors:** Sarah Aldobeaban, Bandr Mzahim, Abdussalam Ali Alshehri

**Affiliations:** 1 Princess Nourah bint Abdulrahman University, Riyadh, Saudi Arabia; 2 King Fahad Medical City, Riyadh, Saudi Arabia

**Keywords:** Metformin, Hypoglycemia, Lactic acidosis

## Abstract

**Background:**

Metformin toxicity is well known to cause lactic acidosis. Multiple cases of hypoglycemia due to isolated metformin overdose have been reported. Increased glucose consumption secondary to anaerobic metabolism has been reported as a possible explanation.

**Case presentation:**

A 23-year-old Arabic woman took 30 g of metformin. In the emergency department, 4 hours after of the event, she was fatigued but vitally stable. During her hospitalization, she had severe lactic acidosis, hypotension corrected with fluid boluses and vasopressors, and multiple episodes of hypoglycemia (6.3 mg/dL, 38 mg/dL, and 42 mg/dL), requiring multiple 50% dextrose-water boluses. The three hypoglycemic episodes occurred coincident with severe lactic acidosis. She improved after 24 hours of continuous renal replacement therapy.

**Conclusions:**

Hypoglycemia can be induced by metformin toxicity in the absence of co-ingestants. A possible explanation of metformin-induced hypoglycemia is increased glucose consumption due to anaerobic metabolism, decreased oral intake, decreased liver glucose production, and decreased glucose absorption.

## Background

Metformin is a biguanide derivative that controls glucose levels through gluconeogenesis reduction and glycogen breakdown inhibition [1]. It also prevents hyperglycemia by reducing gastrointestinal tract absorption of glucose as well as increasing insulin signaling and utilization of glucose [2]. In addition, metformin inhibits the activity of mitochondrial glycerol 3-phosphate dehydrogenase enzyme, which decreases the production of glucose from lactate and glycerol [3]. Accidental and intentional metformin overdoses are commonly reported. In metformin toxicity, lactic acidosis is the most common serious adverse event [4–8].

However, in diabetic patients on metformin monotherapy, cases of hypoglycemia have been reported [9]. It has also been noticed in animal studies with therapeutic doses of metformin [10]. In a 5-year chart review of metformin exposure cases that were reported to the American Association of Poison Control Center (AAPCC), hypoglycemia was reported in 112 (2.8%) of 4072 cases and it was referred to decreased caloric intake, heavy exercise, or sulfonylurea co-ingestion [11]. There are multiple reports of metformin overdose-induced hypoglycemia. However, definitive exclusion of sulfonylurea co-ingestion or insulin use were lacking [12, 13]. One case report of metformin toxicity reported recurrent hypoglycemia where co-ingestion of sulfonylurea or insulin use has been ruled out by extensive laboratory tests [14]. We report a case of metformin toxicity in a young girl who had recurrent hypoglycemic episodes despite the absence of any co-ingestion.

## Case presentation

A 23-year-old Arabic single woman was brought to our emergency department (ED) by her family around 4 hours after intentional metformin ingestion. She was on metformin for weight reduction (her body mass index was 28), as she was found to have polycystic ovarian syndrome (PCOS). She ingested around 60 tablets of 500 mg metformin as a suicide attempt after she experienced a stressful social event. Four years prior, she had undergone a kidney donation to her brother, who had renal failure due to an unknown cause, and otherwise she was healthy. She was not known to have any psychiatric illness or previous suicidal ideation or attempt. There was no history of smoking or alcohol intake. She does not have any family history of diabetes mellitus or mental illnesses. On examination, she was alert and well-nourished but generally fatigued, with no pallor, jaundice, or cyanosis. Her vital signs were as follows: blood pressure 119/65 mmHg, heart rate 122 beat/min, respiratory rate 20 breaths/min, pulse oximetry oxygen saturation 100% on room air, and oral temperature 36.9 °C. She had dry and cool skin, and bilaterally mid-sized pupils, equal and reactive. The rest of her physical examination was unremarkable.

Her bedside point-of-care capillary blood glucose level was checked, and it was low. A peripheral intravenous cannula was inserted, and blood extracted followed by administration of 50 ml (25 g) of 50% dextrose (D50) solution. Her blood glucose level was 6.3 mg/dL in serum chemistry; however, it increased to 106 mg/dL after the D50. After that, 5% dextrose-water solution was initiated as a maintenance infusion. Her blood investigation results are summarized in Table 1. They were unremarkable except for a very low blood glucose level, leukocytosis, hypocalcemia, hyperphosphatemia, and mild creatinine elevation. An initial venous blood gases reading revealed pH: 7.18; PO2: 76.9 mmHg; PCO2: 40.3 mmHg; and bicarbonate of 14.3 mmol/L. Her first lactate level was elevated (8.4 mmol/L), and so a 1 L bolus of Ringer lactate solution was given. Her serial venous blood gases and lactate measurements are shown in Table 2. Results of analyses of her acetaminophen and aspirin levels were negative. In addition, urine analysis as well as urine pregnancy test results were negative.

**Table 1:** Blood investigation results upon emergency department arrival

Test	Value	Normal range
Sodium (mmol/L)	139	136–145
Potassium (mmol/L)	4.5	3.5–5.1
Chloride (mmol/L)	107	95–110
Urea (mmol/L)	3.2	2.5–6.7
Creatinine (umol/L)	124.2	53.0–97.0
Glucose (mg/dL)	6.3	74–106
Albumin (g/L)	40	15–60
Alkaline phosphatase (U/L)	99	40–150
Aspartate transaminase (U/L)	25	5–34
Alanine transaminase (U/L)	16	5–55
Lipase (U/L)	29	8–78
Calcium (mmol/L)	1.28	2.20–2.50
Magnesium (mmol/L)	0.82	0.66–1.07
Phosphate (mmol/L)	2.3	0.74–1.52
Prothrombin time (sec)	16.8	11.5–16.5
Partial thromboplastin time (sec)	32.4	26.0–39.0
International normalized ratio	1.3	0.9–1.2
White blood cell (*10^9/L)	14.8	4.0–11.0
Red blood cell (*10^12/L)	4.52	4.32–5.72
Hemoglobin (g/L)	143.0	135–175
Hematocrit (%)	43	38–46
Mean corpuscular volume (fL)	96.3	80.0–94.0
Mean cell hemoglobin (pg)	31.8	27.0–32.0
Mean cell hemoglobin concentration (g/L)	330.0	320.0–360.0
Platelet (*10^9/L)	361	150–450

**Table 2:** Venous blood gases and lactate levels

	25/4 @ 2058	25/4 @ 2323	26/4 @ 04:05	26/4 @ 10:19	26/4 @ 1347	27/4 @ 0531	27/4 @ 1733
pH	7.18	7.09	7.09	7.33	7.43	7.40	7.38
PCO2 (mmHg)	40.3	28.9	37	33.0	33.6	45.3	36.0
PO2 (mmHg)	76.9	89.0	35	41	93.2	45.9	30.0
HCO3 (mmol/L)	14.3	10.0	11.2	13.3	23.5	26.7	21.3
Lactate (mmol/L)	8.4	12.1	> 13.3	7.7	2.2	0.9	1.0

Two hours later, her capillary blood glucose dropped to 38 mg/dL, and another 50 mL ampule of D50 was infused, which increased her glucose level to 319 mg/dL. During the hospital stay, her blood sugar was monitored frequently (Table 3). As our patient had worsening lactic acidosis, a nephrologist was urgently consulted, and she was admitted to the intensive care unit (ICU). She had a drop in her blood pressure, and so norepinephrine infusion was initiated. After that, continuous renal replacement therapy (CRRT) was started. At around 3 hours later, her blood sugar dropped to 42 mg/dL, and another dextrose bolus was given. After 13 hours of CRRT initiation, the norepinephrine infusion was discontinued, and our patient was hemodynamically stable. The CRRT was continued for 24 hours. Our patient’s renal and liver function tests did not worsen and remained within normal limits till hospital discharge.

**Table 3:** Blood glucose levels (mmol/L)

25/4 @2100	25/4 @2105	25/4 @2230	25/4 @ 23:00	25/4 @2305	25/4 @2345	26/4 @ 0600	26/4 @ 0700	26/4 @ 0800	26/4 @ 0900	29/4 @ 1300
6.3	106	65	38	319	95	127	88	42	145	119

On day 3, she was transferred to the ward with normal mental status and vital signs. She was tolerating oral intake and did not develop any more hypoglycemic attacks. The psychiatrist was consulted for further assessment and treatment. On the fifth day of hospitalization, our patient was discharged home with a good health status. This patient was provided, as a part of our multidisciplinary discharge planning, with follow-up appointments within 1 month for internal medicine, nephrology, and psychiatry. As per our medical records, this patient did not show up for any of these outpatient follow-up appointments.

## Discussion

In this case report, we describe the case of a young woman who developed significant hypoglycemia and a severe lactic acidosis after metformin ingestion. The patient required ICU admission and CRRT. There are numerous publications of metformin-induced hypoglycemia in the medical literature. In most cases the patients had in common that they were suffering malnutrition, performing strenuous exercise, or the patients had comorbidities or other toxic co-ingestions. The patient in our case report was a young woman without previous medical history other than PCOS. Our case is rare in comparison to the other publications about hypoglycemia because our patient was not malnourished and did not have a toxic co-ingestion.

The 2014 annual report of the American Association of Poison Control Centers (AAPCC) reported 8412 cases of biguanide ingestion with 35 major adverse events and 7 deaths [15]. In metformin toxicity, nausea, vomiting, and hyperglycemia were the most commonly reported adverse events, whereas hypoglycemia was reported in only 2% of the cases [16]. Zitzmann *et al.* reported a case of hypoglycemia in an elderly diabetic woman using therapeutic doses of metformin and ACE inhibitors, although her poor nutritional status was a concern [17]. In addition, hypoglycemia following metformin overdose was reported in a 43-year-old woman, and the authors considered that the hypoglycemia was secondary to a net result of metformin toxicity and decreased oral intake with renal impairment, however, co-ingestions were not excluded [12].

A case of hypoglycemia secondary to metformin overdose and kerosene co-ingestion was reported by Rathnapala *et al.* hypothesizing synergism, however, insulin use, or sulfonylurea co-ingestion could not be ruled out [13]. Other reported cases of metformin-related hypoglycemia were associated with heavy exercise, poor oral intake, sulfonylurea co-ingestion, or other comorbidities [18–20].

**Fig. 1:**
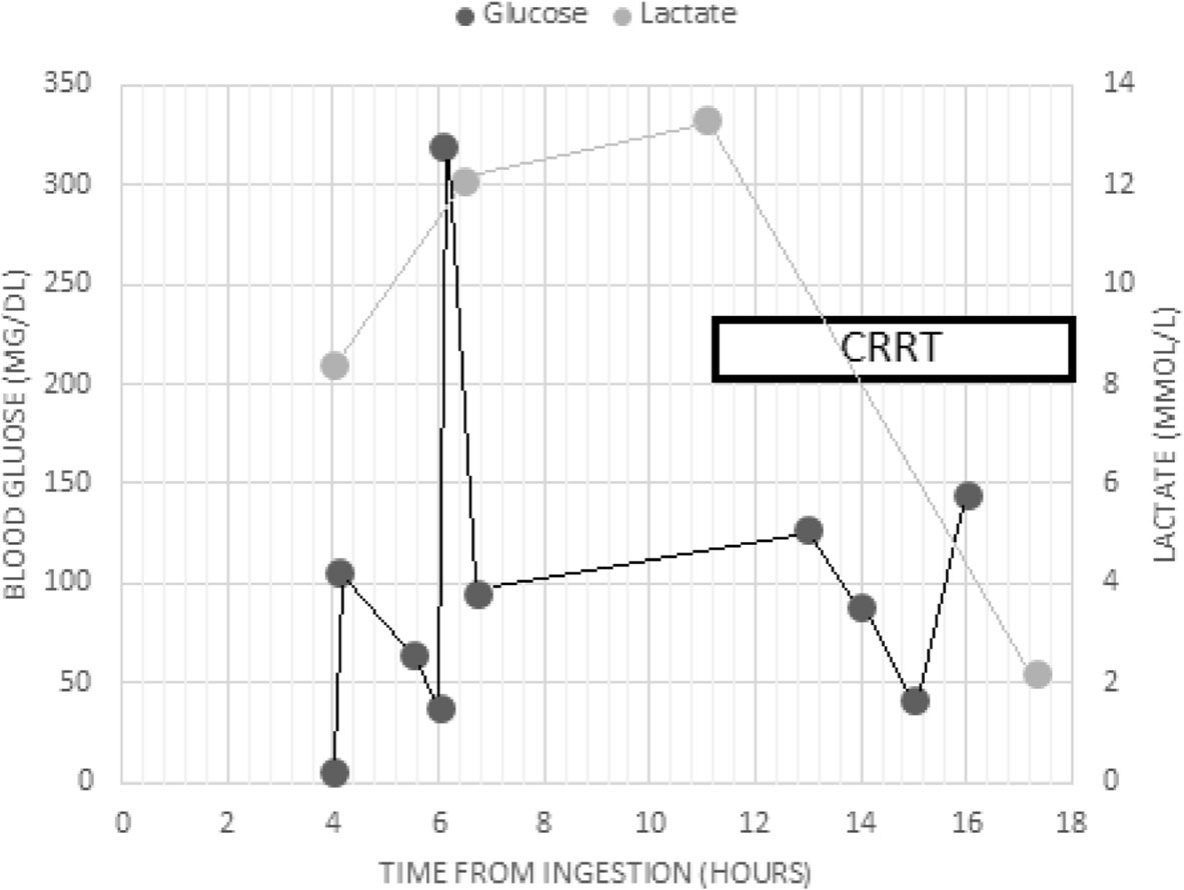
Measurements of blood glucose and lactate levels. *CRRT* continuous renal replacement therapy

However, one case report of two episodes of hypoglycemia secondary to metformin toxicity was a previously healthy patient, who had normal nutritional status, and co-ingestion of sulfonylurea and insulin use was excluded by extensive laboratory tests [14]. Although, one explanation of the hypoglycemia was the increased consumption of glucose due to anaerobic metabolism that happened when lactic acidosis was at its peak [21]. Our patient developed three episodes of hypoglycemia that were corrected with dextrose boluses; however, these episodes were coincidental with severe lactic acidosis. (Fig. 1).

The peak level of lactate occurred before the CRRT was initiated. Our patient was responding very well to the CRRT, her lactic acidosis was resolving with time and her renal function remained intact during her hospital stay. In addition, the norepinephrine infusion was discontinued during CRRT with normal hemodynamic status.

Metformin absorption, liver uptake, and kidney elimination are affected by organic cation transporters (OCTs), with variation in liver uptake and clinical effects due to the differences in their expression level in the liver [22–24]. Some drugs such as rifampin, may increase the metformin effects in decreasing glucose by affecting the OCTs action [25]. OCTs genetic polymorphism or existence of drugs which can affect them might explain the metformin-induced hypoglycemic cases [26]. Our patient strongly denied co-ingestion, however, it was not ruled out completely by advanced laboratory tests.

## Conclusions

Hypoglycemia can be induced by metformin toxicity in the absence of other co-ingestions, and close monitoring of blood glucose is crucial. One possible explanation of metformin-induced hypoglycemia is increased glucose consumption secondary to anaerobic metabolism, however, other mechanisms like decreased oral intake, decreased liver glucose production, and decreased glucose absorption are of consideration.

## Abbreviations

AAPCC: American Association of Poison Control Centers
CRRT: Continuous renal replacement therapy
D50: 50% dextrose
ED: Emergency department
ICU: Intensive care unit
OCTs: Organic cation transporters
